# Domain-General Neural Effects of Associative Learning and Expectations on Pain and Hedonic Taste Perception

**DOI:** 10.1523/JNEUROSCI.1103-25.2025

**Published:** 2025-12-10

**Authors:** Yili Zhao, InSeon Lee, Margaret Rose-McCandlish, Qingbao Yu, Dominik Mischkowski, Jason A. Avery, John E. Ingeholm, Richard Reynolds, Gang Chen, Lauren Yvette Atlas

**Affiliations:** ^1^National Center for Complementary and Integrative Health, National Institutes of Health, Bethesda, Maryland 20892; ^2^Department of Science in Korean Medicine, Graduate School, Kyung Hee University, Seoul 02447, Republic of Korea; ^3^School of Science, Indiana University Indianapolis, Indianapolis, Indiana 46202; ^4^Department of Psychology, University of Illinois, Champaign, Illinois 61820; ^5^National Institute of Mental Health, National Institutes of Health, Bethesda, Maryland 20892; ^6^National Institute on Drug Abuse, National Institutes of Health, Baltimore, Maryland 21224

**Keywords:** anterior insula, associative learning, expectation, orbitofrontal cortex, pain, tastes

## Abstract

Predictive cues significantly influence perception through associative learning. However, it is unknown whether circuits are conserved across domains. We investigated how associative learning influences perceived intensity and valence of pain and hedonic taste and whether expectancy-based modulation varies by aversiveness or modality. Sixty participants (37 females, 23 males) were randomly assigned to receive either painful heat, unpleasant liquid saline, or pleasant liquid sucrose during fMRI scanning. Following conditioning, cues initially associated with low- or high-intensity outcomes were intermittently followed by stimuli calibrated to elicit medium-intensity ratings. Learned cues modulated expectations and subjective outcomes similarly across domains. Consistent with this, the orbitofrontal cortex exhibited domain-general anticipatory activation. Cue effects on perceived intensity and valence were mediated by the left anterior insula and thalamus, respectively—regions closely overlapping those identified in prior studies of pain expectancy (Atlas et al., 2010). Pain specificity was evident when we measured variations in stimulus intensity, whether we used univariate or multivariate approaches, but there was minimal evidence of specificity by modality or aversiveness in cue effects on medium trials. These findings suggest that shared neural circuits mediate the effects of learned expectations on perception, linking pain with other areas of affective processing and perception across domains.

## Significance Statement

Learned expectations shape how we perceive the world, but it remains unclear whether similar brain circuits mediate expectation effects across aversive and hedonic domains. Using single-trial fMRI, we show that predictive cues alter perceived intensity and valence of pain and both aversive and appetitive tastes through shared neural mechanisms. The orbitofrontal cortex, anterior insula, and thalamus supported domain-general modulation, while pain-specific effects emerged primarily when actual stimulus intensity varied. These findings reveal that associative learning engages overlapping neural pathways to influence perception across different sensory and affective experiences, suggesting a unified framework for understanding how the brain constructs subjective experience from expectation.

## Introduction

Associative learning shapes expectations and perception across domains. The orbitofrontal cortex (OFC) is critical for generating and updating reward predictions (O’Doherty, 2004; Rudebeck and Murray, 2014). The OFC and broader ventromedial prefrontal cortex (VMPFC) provide a “common currency” of value (Levy and Glimcher, 2016) to compare outcomes and maximize reward. Although most studies focus on reward, the OFC is also fundamental for aversive learning, including expectations about pain (Roy et al., 2012; Atlas and Wager, 2014; Motzkin et al., 2023). Other valuation regions including the striatum and amygdala are also involved in learning about both appetitive and aversive outcomes, including pain (Seymour et al., 2005; Delgado, 2007; Atlas et al., 2016).

Once associations form, cues prompt predictions that can alter perception across domains, including pain (Atlas and Wager, 2012; Fields, 2018), taste (Nitschke et al., 2006), vision (Balcetis and Dunning, 2006), and others (Kirsch, 1997; Heinz et al., 2013). Only a few neuroimaging studies have compared expectancy effects across domains. Using within-subject designs, Sharvit et al. (2018) and Fazeli and Büchel (2018) crossed pain-predictive cues with cues for other aversive outcomes (disgusting odors or negative pictures, respectively). Both found domain-general expectancy signals in anterior insula and pain-specific responses in the posterior insula (Sharvit et al., 2019). Thus, the insula complements the OFC in processing domain-general expectancy effects. The thalamus also contributes to cue-based expectancy effects on pain and hedonic taste (Nitschke et al., 2006; Atlas et al., 2010), and meta-analyses implicate all three regions in pain expectancy (Atlas and Wager, 2014). Notably, most expectancy studies present explicit cues and/or verbal instructions (Schwarz et al., 2016; Fazeli and Büchel, 2018; Sharvit et al., 2018; Atlas, 2019; Tu et al., 2020) rather than examining learning from cues alone (Jensen et al., 2012, 2015), raising the question of whether mechanisms vary across domains when expectations arise purely through association. Moreover, as prior work on cross-modal expectancy effects was limited to aversive stimuli, it is unknown whether findings extend to appetitive outcomes. We therefore compared appetitive and aversive processing and measured both subjective intensity and valence.

We asked whether the brain mechanisms underlying expectancy effects on aversive and appetitive outcomes are domain-general or domain-specific. We focused particularly on pain, given extensive research on placebo and expectancy effects in this domain, and compared pain with hedonic taste since both are impacted by predictive cues (Nitschke et al., 2006; Atlas and Wager, 2014), have sensory and hedonic qualities, and have overlapping neural activations, particularly in the insula, which contains the gustatory cortex (Corradi-Dell’Acqua et al., 2016; Avery et al., 2020). We applied two established neural patterns that support pain-specific activity and are modulated by expectancy. The neurologic pain signature (NPS; Wager et al., 2013) is sensitive and specific to nociceptive pain, while the stimulus-intensity–independent pain signature (SIIPS; Woo et al., 2017) predicts subjective pain independent of stimulus intensity. We also included a recent domain-general pattern (Čeko et al., 2022) that predicts unpleasantness across aversive, but not appetitive, stimuli. We used these signatures to test whether associative learning and expectancy modulate domain-specific and domain-general processes and to evaluate signature performance based on modality and valence.

**Figure 1.**
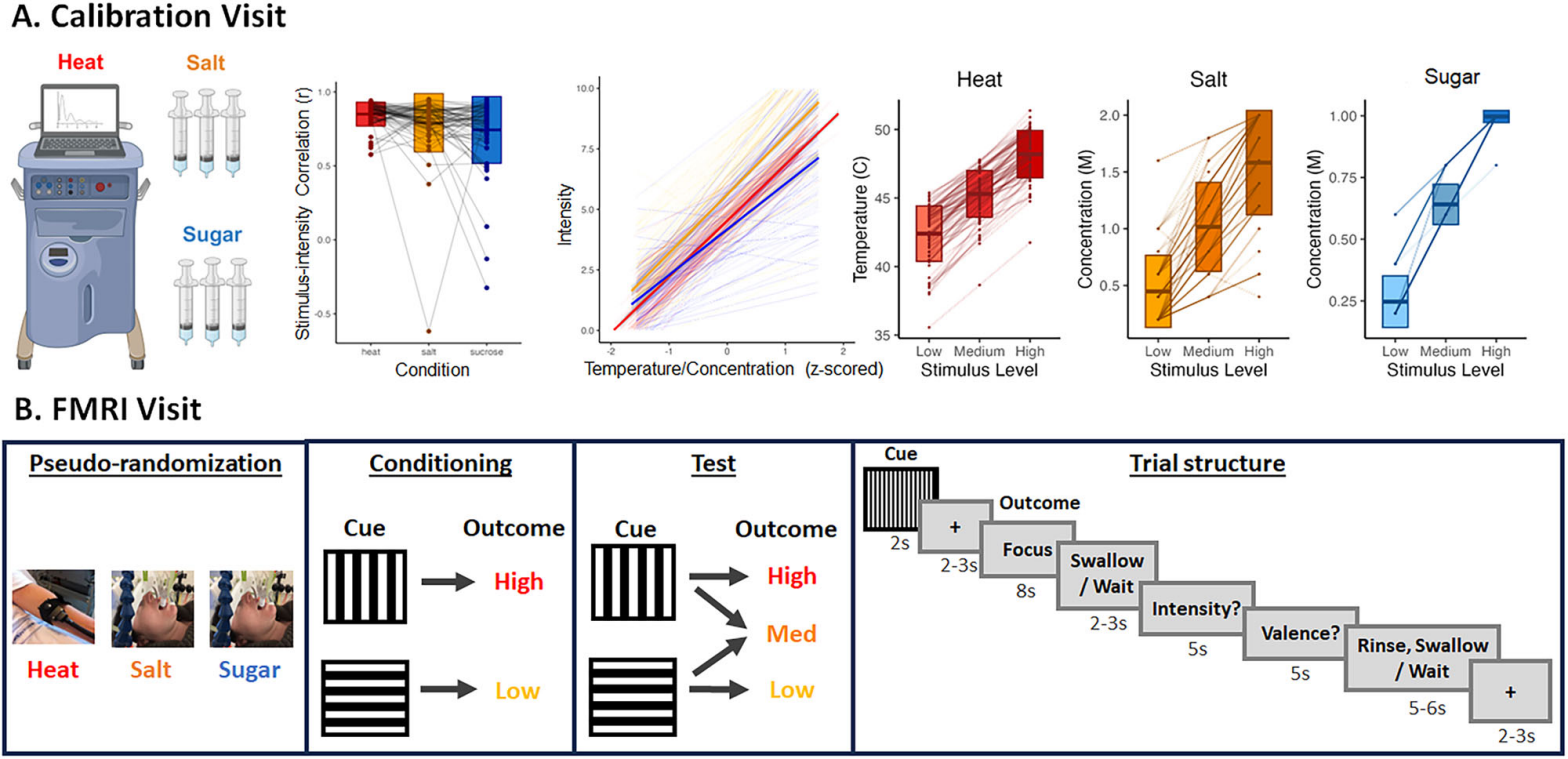
Calibration and fMRI experiment design. ***A***, Calibration design and results. During the calibration visit, all participants underwent thermal stimulation on their left volar forearms and self-administered salt and sucrose solutions at varying intensities and concentrations to evaluate psychophysical profiles for each modality. Stimulus magnitudes were correlated with subjective intensity in each modality (second-left panel). Linear mixed models (middle panel) revealed strong effects of stimulus magnitude (temperature/concentration) on subjective intensity across all modalities (*B* = 2.20; *p* < 0.001), with steepest slopes in the Salt group. For each participant and each modality, temperatures or concentrations were selected corresponding to low, medium, or high intensity for use during the fMRI session (three right panels). ***B***, fMRI experiment design. After the calibration visit, participants were pseudorandomly assigned to one of three groups: Heat, Salt, or Sugar (see Materials and Methods). They returned for an fMRI session, where they underwent a second calibration followed by the fMRI experiment. fMRI scanning began with a conditioning phase, during which High Cues were always followed by a stimulus calibrated to elicit ratings of high intensity, and Low Cues were followed by a stimulus calibrated to elicit ratings of low intensity. Following conditioning, High and Low Cues each had a 50% chance of being followed by a high or low stimulation and a 50% chance of being followed by a stimulus calibrated to elicit moderate intensity. After 8 s, participants in the Salt and Sugar groups were prompted to swallow the solution. Participants reported intensity and valence ratings on each trial. Taste group participants rinsed with a neutral solution between each trial to prevent carry over effects. Heat participants were prompted to wait during the swallow and rinse phases.

We paired predictive cues with painful heat, aversive taste (saline), or appetitive taste (sucrose) during fMRI scanning (Fig. 1). Our between-group design avoided cross-modal comparisons while isolating processes related to learning and expectancy. We asked whether cues modulated expectancy, perception, and brain responses similarly across domains based on modality (pain vs taste) and valence (appetitive vs aversive). We evaluated pain-specific and domain-general neural patterns and tested whether cue effects on intensity and valence were mediated through similar or distinct mechanisms. We preregistered the hypothesis that our task would engage three dissociable circuits: (1) nociception-specific networks sensitive to temperature but not cues or taste (posterior insula, secondary somatosensory cortex); (2) circuits sensitive to modality and expectancy (anterior insula); and (3) domain-general networks that support learning and expectancy across domains (OFC, VMPFC, amygdala, striatum, dorsolateral prefrontal cortex).

## Materials and Methods

The study was approved by the National Institutes of Health Institutional Review Board (Protocol 15-AT-0132; ClinicalTrials.gov Identifier: NCT02446262) and followed the Declaration of Helsinki guidelines for ethical conduct of human research. Analyses were preregistered through Aspredicted (https://aspredicted.org/GXN_FYL).

### Participants

Healthy volunteers from a community sample were screened for this two-visit study. Potential volunteers were deemed ineligible if they had a history of sensory, neurological, or psychiatric disorders, substance abuse, or any major medical condition that could affect somatosensation. Additionally, volunteers who regularly took medication known to affect pain or heat perception were excluded. Eligible volunteers were between the ages of 18 and 50, right-handed, and not pregnant and did not have any condition or device that would pose a risk during the fMRI portion of the study. Participants were also required to have abstained from recreational drugs for the past month and from pain relievers within a time frame equivalent to five half-lives. Seventy-eight participants provided consent during an initial screening and calibration visit (see Procedure in Fig. 1*A*). Sixty participants (37 females; age, 29.58 ± 8.57 years) were determined to be eligible and subsequently completed fMRI scanning on a follow-up visit.

### Materials

#### Thermal pain stimulation

A Medoc Pathway Pain and Sensory Evaluation System (Medoc Advanced Medical Systems) with the Medoc Station software was used to deliver thermal stimuli to participants’ left volar forearm. We used an Advanced Thermal Stimulator thermode with a square contact area of 16 × 16 mm^2^. As addressed below, thermal stimuli lasted for 8 s (1.5 s ramp up, 5 s at peak, 1.5 s ramp to the baseline) and ranged from 32 to 50°C.

#### Appetitive and aversive tastants

We administered liquid tastants to all participants during the calibration visit and to Sugar and Salt groups during the fMRI visit. Sterile solutions were prepared by the NIH Compounding Pharmacy. A combination of potassium chloride and sodium bicarbonate (2.5 mm NaHCO_3_ + 25 mm KCl; 1 ml per trial), which mimics saliva, was used as the neutral solution, consistent with previous taste-fMRI studies (Simmons et al., 2013; Avery et al., 2020). Liquid sodium chloride ranging from 0.2 to 2 M (0.5 ml per trial) was used as the aversive solution. Liquid sucrose ranging from 0.2 to 2 M (0.5 ml per trial) was used as an appetitive solution. During the calibration visit, participants self-administered tastants using syringes. During the fMRI visit, a custom-built pneumatically controlled gustometer (Simmons et al., 2013; Avery et al., 2020) was used to precisely deliver pressurized tastants through intravenous tubing to a 3D-printed mouthpiece. Because we used intravenous tubes for tastant delivery, we used a maximum of 1 M sucrose solution during the fMRI visit as higher concentrations were more viscous and thus did not move smoothly through the tubing. On each visit, we ensured that participants did not consume >1,400 mg of sodium (salt), in accordance with the Food and Drug Administration's recommended daily allowance, and instructed participants to reduce sugar and salt intake for the rest of the day to stay within World Health Organization guidelines.

#### Task delivery and ratings

Stimuli were presented using Psychtoolbox (http://psychtoolbox.org/) and synched with painful stimulation and taste delivery using a LabView (National Instruments) program. During the calibration visit, participants provided verbal ratings of intensity, unpleasantness, and pleasantness using a 0–10 continuous visual analog scale (VAS), where 10 was defined as most intense/unpleasant/pleasant sensation imaginable. We computed the mean intensity and valence [(unpleasantness − pleasantness) for heat and salt; (unpleasantness − pleasantness) for sugar] for use in linear regressions to select temperatures and concentrations for the subsequent fMRI visit. During the fMRI experiment, participants provided ratings using an MRI-compatible trackball (Current Designs). Intensity was again rated on a 0–10 VAS, while valence was measured on a bivalent scale ranging from −10 (most unpleasant imaginable) to 10 (most pleasant imaginable).

### Procedures

#### Day 1: calibration visit

Following an initial phone screen, volunteers deemed potentially eligible were invited to complete a calibration visit (Fig. 1*A*) at the NIH Outpatient Clinic. Seventy-eight participants provided informed consent and underwent a nursing evaluation and a medical exam if they had not had one within the prior year. Seven participants were determined to be ineligible. Following consent and confirmation of eligibility, 71 participants completed questionnaires (which are outside the scope of the current study and may be analyzed in future research; Text S1), followed by a calibration procedure in which they rated the intensity and valence of varying levels of heat, salt solution, sucrose solution, and neutral solution in a counterbalanced order. Calibration procedures were adapted from an adaptive staircase pain calibration procedure we had used previously (see Amir et al., 2022 for complete details). In brief, on each trial for each modality, participants experienced the stimulus and rated its intensity, pleasantness, and unpleasantness following offset using a 0–10 VAS. Each VAS included the following anchors: 0 (no sensation), 5 (moderate intensity/pleasantness/unpleasantness), and 10 (most intense/pleasant/unpleasant sensation imaginable). When rating heat stimuli intensity, participants were also instructed that 1 corresponded to nonpainful warmth, 2 corresponded to the beginning of pain sensation (i.e., pain threshold), and 8 corresponded to highest tolerable pain (i.e., pain tolerance), consistent with prior work (Atlas et al., 2010; Amir et al., 2022). Heat temperatures were selected iteratively based on an iterative regression of temperature and intensity across 24 trials, consistent with previous work (Amir et al., 2022). Tastant concentrations were prepared in advance by the NIH Pharmacy. We tested 11 concentrations varying from 0 to 2 M for salt and sugar (22 trials total) and 5 concentrations for the neutral solution varying from 0 to 100% in 25% increments (10 trials total). Following the calibration, we evaluated the overall correlation between the stimulus level and perception based on intensity ratings. Consistent with previous work (Atlas et al., 2010; Amir et al., 2022), participants were only eligible to continue with a given modality if their correlation coefficients (*r*^2^) between stimulus intensity and perceived intensity exceeded 0.4. For each participant and each modality, we applied a regression model that related stimulus temperature or concentration with the mean intensity/valence to determine stimulus levels corresponding to low (2), medium (5), and high (8) intensity and valence perception (Fig. 1*A*). Detailed results from this procedure are provided in Text S2. These individual levels were used during the fMRI visit. As we could not deliver higher than 1 M sucrose through the intravenous tubing, maximum sucrose concentrations were adjusted (i.e., if the high intensity exceeded 1 M, participants received 1 M sucrose as their maximum concentration). Finally, for neutral solutions, we selected the concentration whose mean intensity was closest to 0.

#### Day 2: fMRI visit

Following the calibration visit, eligible participants were pseudorandomly assigned to one of three task groups (Heat, Salt, or Sugar; Fig. 1*B*). Participants were excluded from Sugar or Salt groups if they perceived salt as pleasant or sugar as unpleasant, respectively, and from the Heat group if their tolerance exceeded safe limits (Text S1). Group assignment was done prior to the fMRI visit so that the NIH Pharmacy could prepare tastants in advance using the concentrations from the calibration visit. Upon arrival, participants provided informed consent for fMRI scanning, confirmed continued eligibility, and completed a set of clinical visit and state assessment questionnaires that may be analyzed in future research. Participants then underwent a follow-up calibration to verify the individual threshold and tolerance levels. Complete details can be found in Text S1.

Following calibration, participants completed a short practice task and were then situated in the MRI scanner and underwent structural scans followed by five runs of fMRI scanning (12 trials per run). All participants were instructed to focus on the relationship between the visual cue and the intensity of the following stimulus. As shown in Figure 1*B*, participants first underwent conditioning (10 trials) during which one cue was followed by a stimulus calibrated to elicit high-intensity ratings (Level 8; “High”; *M*_Heat_ = 48.66°C; SD_Heat_ = 1.35; *M*_Salt_ = 1.71 M; SD_Salt_ = 0.33; *M*_Sugar_ = 0.99 M; SD_Sugar_ = 0.04) and a second cue was followed by a stimulus calibrated to elicit low-intensity ratings (Level 2; “Low”; *M*_Heat_ = 42.87°C; SD_Heat_ = 1.44; *M*_Salt_ = 0.35 M; SD_Salt_ = 0.18; *M*_Sugar_ = 0.27 M; SD_Sugar_ = 0.12). During a subsequent test phase (50 trials), each cue was followed by either the conditioned level or a stimulus calibrated to elicit ratings of medium intensity (Level 5; “Medium”; *M*_Heat_ = 45.86°C; SD_Heat_ = 1.18; *M*_Salt_ = 1.01 M; SD_Salt_ = 0.31; *M*_Sugar_ = 0.66 M; SD_Sugar_ = 0.09), consistent with previous work (Atlas et al., 2010). As shown in Figure 1*B*, each trial began with a 2 s visual cue (counterbalanced across participants) followed by a 2–3 s jittered delay, during which a fixation cross was displayed. The 8 s heat or tastant outcome stimulus was then delivered via the thermode or gustometer while the word “Focus” was presented on the screen. Participants in the Salt and Sugar groups were then prompted to swallow the tastant (“Swallow”) while participants in the Heat group were told to “Wait.” This period lasted 1 s. After a 1–2 s transition, participants used the trackball to rate perceived intensity and valence (5 s, respectively). Missing trials (mean per subject < 13%) were omitted from behavioral analyses and single-trial fMRI analysis. Following a 1–2 s jittered delay, participants in the Salt and the Sugar groups received 1 ml of the neutral solution (*M*_Salt_ = 73.75% concentration; SD_Salt_ = 28.65; *M*_Sugar_ = 73.75% concentration; SD_Sugar_ = 30.65) and were asked to rinse and then swallow the liquid. Participants in the Heat group saw the word “Wait” during this delay. A 2–3 s fixation cross was presented prior to the next trial. Participants rated expected intensity and valence in response to each cue at the end of each run to assess whether uninstructed associative learning resulted in the formation of explicit expectations. However, we did not include expectancy ratings on every trial to avoid drawing attention to expectations which has been shown to alter the timecourse of learning (Atlas et al., 2022b). See Design and Procedure in Figure 1*B*. After the fMRI scan, participants completed post-task questions/questionnaires regarding perception during the scan and in general (Text S1) and then were debriefed and dismissed.

### Behavioral data analysis

Behavioral analyses were conducted using Lme4 (https://cran.r-project.org/web/packages/lme4/index.html) in R version 4.2.1 (https://www.r-project.org/). We analyzed both expectancy ratings and ratings in response to stimulation and separately analyzed intensity and valence ratings. Consistent with our preregistration, our main models included fixed effects for Cue, Stimulus Level, Time (trial for stimulus ratings, block for expectancy ratings), and Group (Heat, Salt, and Sugar; dummy-coded). Follow-up analyses were restricted to medium trials. We also conducted exploratory analyses that modeled Modality (Heat vs Tastes) and Aversiveness [(Heat and Salt) vs Sugar] rather than Group as second-level factors. Group was dummy-coded; Aversiveness, Modality, Cue, Stimulus Level, and Time were mean-centered. In particular, mean centering of Aversiveness and Modality controlled for the unequal number of trials across levels (weights, 1.333 for heat; 0.667 for taste/salt and sugar; 1.333 for appetitive/sugar; 0.667 for aversive/salt and heat). This procedure ensured that any significant effects were not driven by unequal sample sizes. Model comparison was used to evaluate goodness of fit and specification of random factors including both random intercepts and slopes for Stimuli, Cue, Time, and Subject and their interactions. We performed model comparison in a well-established two–step approach (Peng and Lu, 2012): (1) Fixed effects selection, we fit and evaluated a series of predefined models that included the main effects (as listed above), along with all possible two-way and three-way interaction terms, up to the full model including all predictors and their interactions. At this stage, all models included only a random intercept for subject. (2) Random effects selection, After identifying the best-fitting fixed–effect structure (with random intercept only), we introduced random slopes in a stepwise manner. Specifically, we added random slopes for relevant predictors (as listed above) to determine whether model fit improved. The final winning model was selected based on the lowest Akaike information criterion (AIC), with lower AIC values indicating better model performance. All winning models included a random intercept for each subject. For the full dataset (all trials), the winning models also included random slopes for Cue and Stimuli by subject. For medium trials, the winning models included random slopes for Cue and Trial by subject. For details on model specification and results of model comparison, see Text S1 and the shared file *behavioral_model_comparisons.docx* available at https://osf.io/s9xwh/files/osfstorage.

### fMRI acquisition

fMRI data were collected at the NIMH fMRI Facility at the NIH Clinical Center using a General Electric (GE) 3 Tesla MR-750 scanner with a 32-channel head coil. Images were acquired using a multi-echo echoplanar imaging (EPI) sequence [echo times (TEs), 8.8/21.2/33.6 ms; 35 oblique slices for each echo; slice thickness, 3 mm; in-plane resolution, 3 × 3 mm; square field of view (FOV), 216 mm; bottom-up sequential acquisitions; repetition time (TR), 2,000 ms; flip angle, 75°; voxel size, 3 × 3 × 3 mm; acquisition matrix, 72 × 72]. Each slice was oriented in the axial plane but rotated 30° clockwise relative to the AC-PC line to reduce dropout in the VMPFC (Deichmann et al., 2003). This orientation resulted in some areas at the top of the parietal and occipital lobes being outside the scanning window for many participants, and as such, our results are agnostic as to the role of superficial parietal and occipital lobes. Ultrahigh-resolution T1-weighted magnetization–prepared rapid gradient-echo and proton density sequences were used to provide anatomical references for the fMRI analysis. The parameters for these sequences were axial prescription; 172 slices per slab; slice thickness, 1 mm; spacing between slices, 1 mm; square FOV, 256 mm; image matrix, 256 × 256; TE, 3.504 ms; TR, 7,948 ms; flip angle, 7°; voxel size, 1 × 1 × 1 mm.

### fMRI preprocessing

All three echoes of fMRI data were preprocessed using AFNI (http://afni.nimh.nih.gov/afni) with an optimally combined pipeline implemented using afni_proc.py (Cox, 1996). First, the initial 10 s of data were discarded to ensure the scanner reached a steady state. Next, a despiking interpolation algorithm (3dDespike) was applied to remove transient signal spikes from the EPI data. Slice timing correction was then performed using the 3dtshift function. To address spatial distortion artifacts, we used an EPI scan acquired with reverse blip for correction. Head motion was estimated using the 3dVolreg function. We combined across echoes using a voxelwise linear weighted (optimal) combination to optimize signal-to-noise ratio across brain regions with differing T2* values (Poser and Norris, 2009; Posse, 2012). To enhance the signal-to-noise ratio, we applied a 4 mm full-width at half-maximum Gaussian kernel. Finally, the signal intensity for each volume was normalized on a voxelwise basis to reflect percentage signal change. Anatomical scans were warped to standard MNI space using a nonlinear transformation implemented in AFNI's @SSwarper (https://afni.nimh.nih.gov/pub/dist/doc/program_help/@SSwarper.html). The resulting nonlinear transformation matrices were applied to register subject-level statistical maps to MNI space, preserving the original voxel resolution of 3 × 3 × 3 mm without resampling. Subjects with an average Euclidean-normalized derivative of >0.3 during the task were excluded from the group-level analysis due to excessive head motion. As a result, eight subjects were excluded from the original group of 60 subjects, leaving 52 subjects (18 Heat, 17 Salt, 17 Sugar) for the univariate analysis and multivariate pattern analysis (MVPA). Due to technical issues with our trackball leading to missing ratings, additional five participants were excluded from mediation analyses examining associations with intensity (total *n* = 47, 15 Heat, 17 Salt, 15 Sugar), and additional six participants were excluded from mediation analyses examining associations with pleasantness (total *n* = 46, 14 Heat, 17 Salt, 15 Sugar).

### Subject-level fMRI analyses

#### Hemodynamic response function

To ensure that our models captured brain responses to outcomes regardless of modality, we extracted raw activation from the bilateral middle insula (which should be sensitive to all modalities) and right dorsal posterior insula (which is thought to be specific to pain) and fitted five different hemodynamic response functions (HRFs) available in AFNI (WAV, BLOCK4, BLOCK5, SPMG1, MION) to the outcome-evoked response after preprocessing. For each group and each HRF, we used one-sample *t* tests to evaluate ROI activation to High stimuli, High > Low stimuli, and average response across all stimuli. AFNI's MION HRF was determined to provide the best fit for stimulus-evoked responses across groups in bilateral middle insula regardless of Group or Condition (Fig. S1) and was therefore used in first-level analyses. Complete details are provided in Text S1.

#### General linear model

General linear models (GLM) were implemented using AFNI's 3dDeconvolve (https://afni.nimh.nih.gov/afni/doc/help/3dDeconvolve.html). For each individual, the regression model included regressors for each primary event of interest, which were constructed using the “regress_basis_multi” function in AFNI's 3dDeconvolve program. The present analyses focus on responses to cues, which were modeled using 2 s boxcars convolved with AFNI's default HRF [“BLOCK(2,1)” in AFNI], and responses to stimulus outcomes, which were modeled with an 8 s boxcar, convolved with the MION HRF. We modeled separate regressors for each condition (i.e., High Cue, Low Cue, High Stimulus, Low Stimulus, High Cue + Medium Stimulus, Low Cue + Medium Stimulus). We also included a 1 s event at stimulus offset to model swallow-related activity, a 10 s boxcar to model the rating period, and a 4 s boxcar to model the rinse period; these were not analyzed in the present study. Motion parameters were incorporated as covariates of no interest to account for potential confounding effects.

#### Single trial analyses

Single-trial analyses require estimates per trial, rather than per condition. Whole-brain trial–level estimates were generated using AFNI's 3dREMLfit program (https://afni.nimh.nih.gov/pub/dist/doc/htmldoc/statistics/remlfit.html). We included the same regressors of no interest and cues as the GLM but modeled each outcome trial (i.e., heat or tastant delivery) separately with its own 8 s MION HRF. This generates a coefficient per voxel per trial which was used as a measure of trial-evoked activation and used in voxelwise multilevel mediations, described in more detail below.

### Group-level fMRI analyses

#### Mass-univariate analyses

For group-level analysis, we initially performed a full factorial design with Statistical Parametric Mapping (SPM12; https://www.fil.ion.ucl.ac.uk/spm/software/spm12/) to explore brain activation associated with varying stimuli intensities. Six contrasts were constructed, comparing responses to stimulus outcome as a function of Level (High vs Low) for Heat, Salt, and Sugar, in both directions. Medium stimuli were excluded from this analysis to prevent confounding from cue effects, which could obscure activations attributable solely to stimulus level (i.e., changes in temperature or concentration). Subsequently, another full factorial design was employed to examine activations during anticipation as a function of Cue (High vs Low), considering both directions and analyzing both across and within groups. A conjunction analysis was conducted to identify shared neural activations across all three groups and each pair of groups for each contrast.

#### MVPA

We used MVPA implemented in the Decoding Toolbox (https://sites.google.com/site/tdtdecodingtoolbox/) via MATLAB to further validate the activity identified in the univariate analysis. Group-level analysis was conducted using SPM. We performed a one-sample *t* test to evaluate the whole-brain patterns capable of differentiating between High and Low cues with >50% accuracy, based on individual accuracy maps extracted from all participants. For additional information, see subject-level MVPA analysis in Text S1.

#### Multilevel mediation and moderation analyses

Voxelwise multilevel mediation was implemented in MATLAB using the M3 toolbox, which implements linear mixed models [for complete details, see Wager et al. (2008, 2009); Atlas et al. (2010)]. We used multilevel mediation analysis to identify neural activations that explained cue effects on subjective perception in response to medium stimuli and applied moderated mediation to determine whether effects differ across groups. Consistent with previous work (Atlas et al., 2010, 2021), our mediation models designated variations in Cue (High–Low) as the input variable (*X*), ratings on medium trials as the outcome variable (*Y*), and voxelwise activation on medium trials as the potential mediator (*M*). Trial-by-trial voxelwise activation was operationalized using single-trial estimates from AFNI's 3dREMLfit as described above. Because single-trial estimates are sensitive to noise, we evaluated variance inflation factors (VIFs) in a design matrix incorporating trial estimates and nuisance regressors and removed any trials whose VIFs exceeded 2 prior to analysis, consistent with prior work (Atlas et al., 2010, 2014, 2022a). As with prior work (Atlas et al., 2010), Path *a* in our mediation framework captures the effect of predictive cues on brain activation (*X*–*M*), Path *b* evaluates the association between activation and rating when controlling for cue (*M*–*Y*), and the mediation effect tests the indirect effect of cues on ratings through changes in brain activation or the difference between the total effect (effect of *X* on *Y*) and the direct effect when controlling for the mediator. In multilevel mediation, the mediation effect (*c*–*c*’) is equivalent to the product of Paths *a* and *b* plus their covariance (i.e., *c*–*c*’ = a*b + cov(*a*,*b*); Kenny et al., 2003). We thus extracted responses from mediators and inspected outcomes to determine whether they were driven by consistent responses or variations across individuals. Significance was tested using bootstrapping to account for the covariance between Paths A and B.

We separately searched for brain mediators of cue effects on subjective intensity and cue effects on subjective valence. Our intensity mediation treated Cue [High > Low] as the input variable for all groups. However, since high cues were associated with the more unpleasant outcome for Heat and Salt groups, whereas they were associated with the more pleasant outcome for the Sugar group, we accounted for group differences in overall aversiveness in our valence mediation. We accomplished this by reversing the cue contrast depending on stimulus aversiveness: While Path *a* for the Sugar group was the same as in the intensity model (High > Low), Path *a* was coded as (Low > High) for the Heat and Salt groups. Since trial-wise valence/pleasantness ratings served as *Y*, this leads to an overall positive association between *X* and *Y* regardless of the group.

For each mediation, we evaluated mediation regardless of the group and implemented bootstrapping to evaluate significance. We also conducted moderation analyses to explore whether path effects varied as a function of Group [two moderators, (Heat > Salt) and (Salt > Sugar)], Modality (Heat > Taste), or Aversiveness (Appetitive > Aversive) and to evaluate results when controlling for Group/Modality/Aversiveness.

### Statistical thresholding and regions of interest

#### Neural signature patterns

To explore whether pain-specific networks were modulated by stimulus intensity or learned expectations, we used three validated neural signature patterns (NPS; Wager et al., 2013; SIIPS; Woo et al., 2017; and the negative affect pattern, Čeko et al., 2022). We computed the dot product of the pattern weights and neural betas and contrast maps (e.g., High vs Low stimulus level) and performed one-sample *t* tests for each group and used one-way analysis of variance (ANOVA) for each pattern to determine whether there were significant differences in pattern responsiveness across groups. We anticipated identifying a distinct fit of Heat responses within pain-specific neural patterns (NPS and SIIPS) but expected no differences with the aversiveness-general pattern (the negative affect pattern) between Heat and Salt. Multiple comparisons among three groups were adjusted using Bonferroni’s correction. The same approach was used to evaluate whether there were significant effects on signature patterns for each path of our mediation analyses.

#### Regions of interest

Small volume correction was performed for each analysis, using a region of interest (ROI) mask from our previous meta-analysis, which identified placebo-related increased activations (Atlas and Wager, 2014). Consistent with our preregistration, we focus on responses to medium-intensity pain and nonpain stimuli in SI, SII, ACC, insula, operculum, thalamus, periaqueductal gray, striatum, DLPFC, OFC/VMPFC, and the amygdala. Family wise error (FWE) correction (*p* < 0.05) was applied for small volume correction.

#### Univariate and MVPA analyses

For whole-brain exploratory analysis, we applied an initial voxel-level statistical threshold of *p* < 0.001 (uncorrected), with a cluster extent threshold of 10 contiguous voxels to enhance result robustness. We further performed FWE correction (*p* < 0.05) and reports all activations survived this stricter correction.

#### Mediation and moderation analyses

We report mediation and moderation results that survived whole-brain false discovery rate (FDR) correction (*q* < 0.05), since there were no results that survived correction within regions involved in pain and placebo analgesia. Whole-brain uncorrected exploratory results are reported at a voxelwise threshold of *p* < 0.001 with at least three contiguous voxels; clusters were defined based on being contiguous with additional voxels at *p* < 0.005 and *p* < 0.01, consistent with prior work (Atlas et al., 2010).

## Results

### Behavioral outcomes: stimulus level, expectation, and cue effects

#### Effects of the stimulus type and magnitude on subjective perception

We used linear mixed models to examine associations between changes in stimulus magnitude (low, medium, or high temperature or concentration) and subjective perception on the fMRI visit, when subjects were assigned to Group. We focus on the preregistered model that controlled for Group differences (Heat vs Salt; Salt vs Sugar); behavioral results for models that replaced Group with Modality (Heat vs Taste) or Aversiveness (Appetitive vs Aversive) are provided in Table S1 for completeness and are acknowledged when they are inconsistent with our main model.

**Figure 2.**
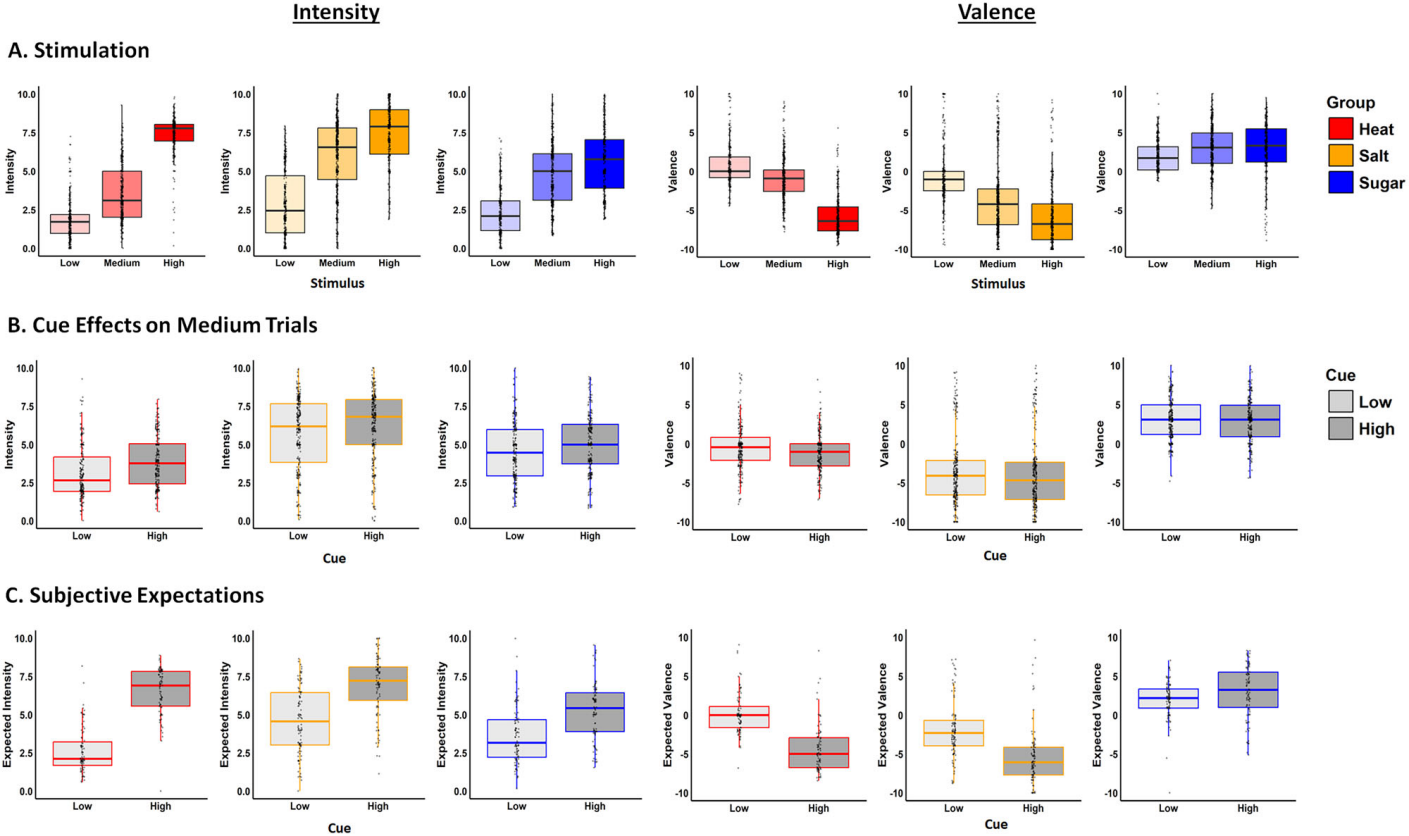
Behavioral results during the fMRI visit. ***A***, Effects of changes in stimulation on subjective intensity (left) and valence (right) for Heat (red), Salt (orange), and Sugar (blue) groups. Intensity ratings increased with stimulus intensity across all groups (main effect, *B* = 1.940; *p* < 0.001), while valence ratings became more negative (unpleasant) as stimulus intensity increased for Heat and Salt (Stimulus level × Group Heat vs Salt, *B* = 0.355; *p* = 0.03) and more positive (pleasant) for Sugar (Stimulus level × Group Salt vs Sugar, *B* = 0.575; *p* < 0.001; Table 1). ***B***, Cue effects on subjective intensity (left) and valence (right). Across groups, participants reported higher intensity and less pleasantness in response to High Cues (dark) than Low Cues (light; intensity, *B* = 0.305; *p* < 0.001; valence, *B* = −0.201; *p* = 0.004; Table 2). There were no interactions with Group. ***C***, Cue effects on subjective expectations. Across all groups, participants anticipated higher intensity (*B* = 1.312; *p* < 0.001) and higher unpleasantness (*B* = −1.187; *p* < 0.001) following High Cues compared with Low Cues. We observed significant interaction between Group and Cue for both intensity and valence expectations, driven by larger cue-induced difference for Heat relative to Salt and for Salt relative to Sugar (Table 3).

We observed significant main effects of Stimulus Level on both intensity and valence, such that participants reported greater subjective intensity and subjective unpleasantness as a function of outcome magnitude across all groups (Fig. 2*A*; Intensity, *B* = 1.940; *p* < 0.001; Valence, *B* = 1.662; *p* < 0.001).

We observed significant main effects of Group in both models, driven by higher-intensity ratings in the Salt group than the Heat group (*B*_Heat vs Salt_ = −1.043; *p* < 0.01; Heat, Mean = 4.161; SD = 2.643; Salt, Mean = 5.546; SD = 2.870) and by higher valence ratings in the Sugar group than the Salt group in valence (*B*_Salt vs Sugar_ = −3.413; *p* < 0.001; Salt, Mean = −3.439; SD = 4.646; Sugar, Mean = 2.698; SD = 2.855). Consistent with these group differences, our additional models (Table S1) confirmed that intensity ratings were impacted by Modality (*B*_Pain vs Tastes_ = −0.785; *p* < 0.001) but not Aversiveness (*p* > 0.50), while valence ratings were impacted by Aversiveness (*B*_Aversive vs Appetitive_ = −2.591; *p* < 0.001), but not Modality (*p* > 0.50). For complete results, see Table 1 and Table S1.

**Table 1.** Results of the winning group models for intensity ratings and valence ratings in all trials

	Estimate	*T* value	*p* value
The winning model: Intensity ∼ Group_Pain vs Salt × Cue × Stimuli × Trial + ∼ Group_Salt vs Sugar × Cue × Stimuli × Trial + (1 + Stimuli × Cue|Subject)			
Group_Pain vs Salt	−1.043	−3.329	0.002**
Cue	0.309	5.054	<0.001***
Stimuli	1.940	17.556	<0.001***
Trial	0.003	1.181	0.238
Group_Salt vs Sugar	0.149	0.478	0.634
Group_Pain vs Salt × Cue	0.091	1.025	0.310
Group_Pain vs Salt × Stimuli	0.355	2.228	0.030*
Stimuli × Cue	−0.052	−0.416	0.679
Group_Pain vs Salt × Trial	−0.014	−4.382	<0.001***
Cue × Trial	−0.003	−1.341	0.180
Stimuli × Trial	0.009	3.368	<0.001***
Group_Salt vs Sugar × Cue	0.063	0.730	0.468
Group_Salt vs Sugar × Stimuli	0.575	3.670	<0.001***
Group_Salt vs Sugar × Trial	0.001	0.203	0.839
Group_Pain vs Salt × Cue × Stimuli	1.055	5.832	<0.001***
Group_Pain vs Salt × Cue × Trial	−0.001	−0.163	0.871
Group_Pain vs Salt × Stimuli × Trial	−0.003	0.635	0.525
Stimuli × Cue × Trial	0.003	1.239	0.215
Group_Salt vs Sugar × Cue × Stimuli	0.507	2.840	0.006**
Group_Salt vs Sugar × Cue × Trial	−0.006	−1.815	0.070
Group_Salt vs Sugar × Stimuli × Trial	0.008	2.041	0.041*
Group_Pain vs Salt × Cue × Stimuli × Trial	0.005	1.373	0.170
Group_Salt vs Sugar × Cue × Stimuli × Trial	−0.001	−0.259	0.796
The winning model: Valence ∼ Group_Pain vs Salt × Cue × Stimuli × Trial + ∼ Group_Salt vs Sugar × Cue × Stimuli × Trial + (1 + Stimuli × Cue|Subject)			
Group_Pain vs Salt	−0.459	−0.833	0.408
Cue	−0.211	−3.056	0.004**
Stimuli	−1.670	−9.468	<0.001***
Trial	−0.034	−9.952	<0.001***
Group_Salt vs Sugar	−3.413	−6.231	<0.001***
Group_Pain vs Salt × Cue	−0.143	−1.423	0.160
Group_Pain vs Salt × Stimuli	−1.280	−5.039	<0.001***
Stimuli × Cue	−0.748	−2.732	0.008**
Group_Pain vs Salt × Trial	−0.031	6.369	<0.001***
Cue × Trial	−0.0002	−0.083	0.934
Stimuli × Trial	−0.009	−2.050	0.040
Group_Salt vs Sugar × Cue	−0.014	−1.401	0.167
Group_Pain vs Salt × Stimuli	−2.154	−8.614	<0.001***
Group_Salt vs Sugar × Trial	0.004	0.818	0.413
Group_Pain vs Salt × Cue × Stimuli	−0.757	−1.926	0.059
Group_Pain vs Salt × Cue × Trial	0.001	0.215	0.830
Group_Pain vs Salt × Stimuli × Trial	0.007	1.193	0.233
Stimuli × Cue × Trial	0.012	2.825	0.005**
Group_Salt vs Sugar × Cue × Stimuli	−0.079	−0.204	0.838
Group_Salt vs Sugar × Cue × Trial	0.006	1.135	0.256
Group_Salt vs Sugar × Stimuli × Trial	0.001	0.135	0.823
Group_Pain vs Salt × Cue × Stimuli × Trial	−0.009	−1.376	0.140
Group_Salt vs Sugar × Cue × Stimuli × Trial	−0.002	−0.402	0.688

*** 0.001.

** 0.01.

* 0.05.

Main effects of Group and Stimulus Level were qualified by a significant Group × Stimulus Level interaction in all models (all *p*'s < 0.002; Table 1). There were stronger effects of Stimulus Level on intensity in the Salt group than the Heat group (*B*_Heat vs Salt_ = −1. 043; *p* = 0.0016), and there were positive effects of Stimulus Level on valence in the Sugar group and negative effects in the Pain and Salt groups (*B*_Salt vs Sugar_ = −3.413; *p* < 0.001).

Finally, all models revealed a significant main effect of Cue, such that stimuli preceded by high cues were perceived as more intense (High, Mean = 6.169; SD = 2.283; Low, Mean = 3.332; SD = 2.312; all *p*'s < 0.001) and more unpleasant (High, Mean = −1.987; SD = 5.191; Low, Mean = 0.232; SD = 3.827; all *p*'s < 0.001) than stimuli preceded by low cues. We did not observe interactions between Group and Cue or between Group, Cue, and Trial in any model (Table 1; Table S1). Importantly, we did observe significant Group × Stimulus × Cue interactions in the intensity model (*B*_Heat vs Salt_ = 1.055; *p* < 0.001; *B*_Salt vs Sugar_ = 0.507; *p* < 0.01) and a Stimulus × Cue interaction in valence models (*B* = −0.748; *p* < 0.01). We therefore conducted follow-up models restricted to medium trials, which were equally crossed with cues. See all results in Table 1 and Table S1.

#### Learned cues modulate subjective perception in response to medium stimulation

We observed a significant main effect of Cue on Intensity (*B* = 0.301; *p* < 0.001) and Valence (*B* = −0.201; *p* = 0.004), such that medium stimuli preceded by high cues were perceived as more intense and more unpleasant than those preceded by low cues (Mean Intensity, High = 5.184; Low = 4.564; *p* < 0.001; Mean Valence, High = −0.786; Low = −0.435; all *p*'s < 0.01; Fig. 2*B*). There were no interactions between Group and Cue or between Group, Cue, and Trial in any model (none of those interactions survived model comparison and they remained nonsignificant even when included in the models), indicating that learned cues modulated perception similarly across domains.

Consistent with analyses across all trials, we observed significant main effects of Group on intensity ratings (*B*_Heat vs Salt_ = −1.146; *p* < 0.001) and valence ratings (*B*_Salt vs Sugar_ = −3.461; *p* < 0.001) in response to medium stimulation, driven by higher intensity in the Salt group than the Heat group (Heat, Mean = 3.581; SD = 1.783; Salt, Mean = 5.962; SD = 2.359; Table 2) and higher valence ratings in the Salt group than the Sugar group (Salt, Mean = −3.630; SD = 4.608; Sugar, Mean = 2.956; SD = 2.783; Table 2). There were no differences in intensity between Salt and Sugar groups or in valence between Pain and Salt (all *p*'s > 0.40).

**Table 2.** Results of the winning group models for intensity ratings and valence ratings in medium trials

	Estimate	*T* value	*p* value
The winning model: Intensity ∼ Group_Pain vs Salt × Trial + Group_Salt vs Sugar × Trial + Cue + (1 + Cue + Trial |Subject)			
Group_Pain vs Salt	−1.146	−3.764	<0.001***
Trial	0.002	0.600	0.551
Group_Salt vs Sugar	0.036	0.119	0.906
Cue	0.305	5.140	<0.001***
Group_Pain vs Salt × Trial	−0.015	−2.695	0.009**
Group_Salt vs Sugar × Trial	40.0001	0.011	0.991
The winning model: Valence ∼ Group_Pain vs Salt × Trial + Group_Salt vs Sugar × Trial + Cue + (1 + Cue + Trial |Subject)			
Group_Pain vs Salt	−0.373	−0.661	0.511
Trial	−0.033	−4.004	<0.001***
Group_Salt vs Sugar	−3.461	−6.239	<0.001***
Cue	−0.201	−3.013	0.004**
Group_Pain vs Salt × Trial	−0.035	−2.853	0.006**
Group_Salt vs Sugar × Trial	0.006	0.543	0.589

*** 0.001.

** 0.01.

Finally, we observed a main effect of Trial on valence ratings (*B* = −0.036; *p* < 0.001), such that stimuli were perceived as more unpleasant over time. See complete results in Table 2.

Results were similar when we modeled main effects of Aversiveness and Modality (Table S1). Thus, our finding of main effects of Cue on valence and intensity ratings regardless of Group provides behavioral evidence for a domain-general effect of predictive cues on subjective perception across modalities and aversiveness.

To examine whether the observed behavioral relationships related to cue effects were consistent across groups, we conducted correlation analyses between perceived intensity and valence on the medium-intensity trials for each group. Results showed that the overall correlation directions aligned with our expectations: for Heat and Salt, higher expected or perceived intensity was associated with greater unpleasantness (*r* = −0.524; *p* = 0.026; *r* = −0.713; *p* < 0.001, respectively), whereas for Sugar, higher expected or perceived intensity was marginally associated with greater pleasantness (*r* = 0.451; *p* = 0.053). To examine whether the strength of this relationship differed across groups, we conducted Fisher's *z*-transformations to compare the correlation coefficients. The results revealed that the magnitude of the correlations was significantly higher for Heat versus Sugar (*z* = −2.971; *p* = 0.009) and Salt versus Sugar (*z* = −3.960; *p* < 0.001), whereas no difference emerged between Heat and Salt (*z* = 0.879; *p* = 1.000). All comparisons were conducted using Bonferroni-corrected *p* values.

#### Learned cues induce conscious expectations

We also asked whether learned cues influenced participants’ explicit expectations. Note that in the expectation models, we included the time effect of block, as expectations were measured at the beginning of each block. This differs from perceived ratings which were collected on a per-trial basis. When we modeled expected intensity, we observed a significant main effect of Group, driven by higher-intensity ratings in the Salt group than the Sugar group (*B*_Salt vs Sugar_ = 0.516; *p* = 0.018; Salt, Mean = 5.820; SD = 2.296; Sugar, Mean = 4.425; SD = 2.029). We also observed a main effect of Cue (*B* = 1.312; *p* < 0.001), such that participants anticipated a higher intensity in response to High Cues (Mean = 6.253; SD = 1.972) relative to Low Cues (Mean = 3.667; SD = 2.024). This effect was qualified by a significant interaction of Group and Cue, driven by stronger cue-induced differences in expected intensity in the Heat group relative to the Salt (*B*_Heat vs Salt_ = 0.612; *p* < 0.0018) and in the Salt group relative to the Sugar groups (*B*_Salt vs Sugar_ = 0.499; *p* < 0.001; Figure 2*C*).

We observed similar effects when we examined expected valence (Fig. 2*C*). There was a main effect of Group, driven by higher expected unpleasantness in the Salt group compared with the Heat group (*B*_Heat vs Salt_ = −1.021; *p* = 0.019; Salt, Mean = −2.016; SD = 23.781; Sugar, Mean = −3.640; SD = 4.145) and greater pleasantness in the Sugar group compared with the Salt group (*B*_Salt vs Sugar_ = −3.581; *p* < 0.001; Sugar, Mean = 2.492; SD = 2.943). We also observed a main effect of Cue (*B* = −1.187; *p* < 0.001), indicating that participants expected overall greater unpleasantness with the High Cue (Mean = −2.215; SD = 5.102) than with the Low Cue (Mean = 0.105; SD = 3.441), and a significant Group × Cue interaction, characterized by larger cue-induced differences in expected valence when comparing the Heat group to the Salt group (*B*_Heat vs Salt_ = −1.157; *p* < 0.001) and the Salt group to the Sugar group (*B*_Salt vs Sugar_ = −1.722; *p* < 0.001). See complete results in Table 3.

**Table 3.** Results of the winning group models for expected intensity and valence ratings in medium trials

	Estimate	*T* value	*p* value
The winning model: Expected Intensity ∼ Group_Pain vs Salt × Cue + Group_Salt vs Sugar × Cue + Block + (1 + 1 |Subject)			
Group_Pain vs Salt	−0.349	−1.643	0.106
Cue	1.312	20.028	<0.001***
Group_Salt vs Sugar	0.516	2.447	0.018*
Block	0.079	1.701	0.090
Group_Pain vs Salt × Cue	0.612	6.499	<0.001***
Group_Salt vs Sugar × Cue	0.499	5.419	<0.001***
The winning model: Expected Valence ∼ Group_Pain vs Salt × Cue × Block + Group_Salt vs Sugar × Cue × Block + (1 + 1 |Subject)			
Group_Pain vs Salt	−1.021	−2.412	0.019*
Cue	−1.187	−11.642	<0.001***
Block	−0.299	−4.115	<0.001***
Group_Salt vs Sugar	−3.581	−8.513	<0.001***
Group_Pain vs Salt × Cue	−1.157	−7.884	<0.001***
Group_Pain vs Salt × Block	0.365	3.489	<0.001***
Cue × Block	−0.082	−1.131	0.259
Group_Salt vs Sugar × Cue	−1.722	−12.000	<0.001***
Group_Salt vs Sugar × Block	−0.092	−0.900	0.368
Group_Pain vs Salt × Cue × Block	−0.047	−0.452	0.651

*** 0.001.

* 0.05.

Results for models that measure the impact of Modality or Aversiveness rather than Group were consistent with our main model (Table S2). These results underscore the significant role of predictive cues in modulating expectations of both intensity and valence, even without verbal instructions. Together, the behavioral results indicate that associations between predictive cues and the stimuli we administered led to changes in conscious expectations and perception across domains.

Additionally, we conducted single-level correlations between expected ratings and subjective experiences. For both intensity and valence (*N* = 47), we observed significant correlations between expected ratings and perceived ratings of medium-intensity stimuli (Intensity, *r* = 0.582; *p* < 0.001; Valence, *r* = 0.495; *p* < 0.001).

### Neural activation varies as a function of outcome magnitude and modality

#### Comparing high versus low stimuli within each group

We first examined how variations in the stimulus level affected brain responses during outcome delivery separately for each group. MVPA results were largely consistent with univariate results; we therefore focus on univariate results and signature patterns in the main manuscript and report MVPA results in Text 2, Figure S2, and Table S4.

Univariate results in the Heat group survived small volumes correction (SVC) within a priori brain regions associated with pain and placebo analgesia (Table 4). Relative to low heat stimulation, high heat stimulation elicited increases in the anterior and the midcingulate cortex (ACC/MCC), the right supramarginal gyrus, the anterior insula (aIns), the right Rolandic operculum, the posterior insula, bilateral caudate nucleus, the supplementary motor area (SMA), and the frontal gyrus. Whole-brain FWE correction also revealed differences in the left cerebellum (Table 4). Changes in the stimulus level (High > Low) were significantly associated with the expression of all three neural signature patterns (Mean, NPS = 0.987; SD = 0.572; SIIPS = 236.163; SD = 161.649; Negative Affect = 0.042; SD = 0.041; all *t*'s > 3.905; *p*'s < 0.005).

**Table 4.** Results of mass-univariate functional analyses regarding stimulation level for heat, salt, and sugar in the MNI space

Region label	Correction	BA	Cluster size	*x*	*y*	*z*	*t* value
Heat: high–low							
Supramarginal gyrus (R)	SVC^a^	40	33	58	−32	28	6.97
Thalamus (R)	SVC	50	44	2	−10	14	6.66
Caudate (R)	SVC	48		14	−8	20	6.02
Cerebellum (L)	SVC	37	22	−38	−52	−32	6.57
Mid-cingulum (R)	SVC	32	31	2	10	40	6.37
Mid-cingulum (L)	SVC	32		−2	14	34	5.96
Insula (R)	SVC	1	3	34	−20	20	6.1
Rolandic operculum (R)	SVC	6	6	56	2	8	5.89
Insula (L)	SVC	13	7	−40	8	2	5.86
Caudate (L)	SVC	48	3	−10	2	10	5.77
SMA (R)	FWE^b^	6	40	4	−14	62	5.98
Postcentral gyrus (R)	FWE	4	2	26	−26	58	6.02
Cerebellum (L)	FWE	18	3	−4	−70	−44	5.65
SMA (L)	FWE	6	3	−8	−10	74	5.49
Supramarginal gyrus (L)	FWE	39	1	−58	−44	28	5.42
OFC (L)	FWE	10	1	−34	56	−2	5.34
Lingual gyrus (R)	FWE	36	1	14	−26	−8	5.34
Putamen (L)	FWE	49	1	−26	10	4	5.27
Salt: High–low							
Postcentral gyrus (R)	FWE	33	52	−8	32	52	−8
Postcentral gyrus (L)	FWE	10	−50	−10	32	−50	−10
Rectus (L)	FWE	3	−2	62	−16	−2	62
Cerebellum (L)	FWE	2	−26	−62	−20	−26	−62
Cerebellum (R)	FWE	1	16	−50	−50	16	−50

Results are thresholded with ^a^small volume correction (SVC; *p* < 0.05, FWE correction) in the pain-placebo analgesia mask (Atlas and Wager, 2014), or only ^b^cluster-wise FWE correction in the whole brain (FWE). Only the Heat Group survived SVC with the pain-placebo analgesia mask. Only Heat and Salt showed significant whole-brain activations after FWE correction. No corrected results are survived for sugar. BA, Brodmann area; L, left hemisphere; mid, middle; R, right hemisphere; SMA, supplementary motor area; OFC, orbitofrontal cortex.

For salt concentration (High > Low), whole-brain FWE correction revealed robust activations in SII, the middle frontal gyrus, and the cerebellum (Table 4). No voxels survived correction within regions involved in pain and placebo (see Text 2 and Table 3 for whole-brain uncorrected results). Similar to the Heat group, changes in stimulus level ([High > Low]) were associated with significant expression of all three signature patterns (Mean _NPS_ = 0.451; SD _NPS_ = 0.564; Mean _SIIPS_ = 96.512; SD _SIIPS_ = 88.780; Mean _Negative Affect_ = 0.034; SD _Negative Affect_ = 0.062; all *t*'s > 2.312; *p*'s < 0.05).

In contrast to the Heat and Salt groups, we did not observe any significant activations that survived FWE correction when we evaluated how changes in sucrose concentration (High > Low) impacted activation in the Sugar group, nor did we observe significant expression of any signature pattern (all *t*'s < 1.131; *p*'s > 0.2). Results of whole-brain exploratory analyses are reported in Text 2 and Table 4.

#### Comparisons between groups using neural signature patterns

**Figure 3.**
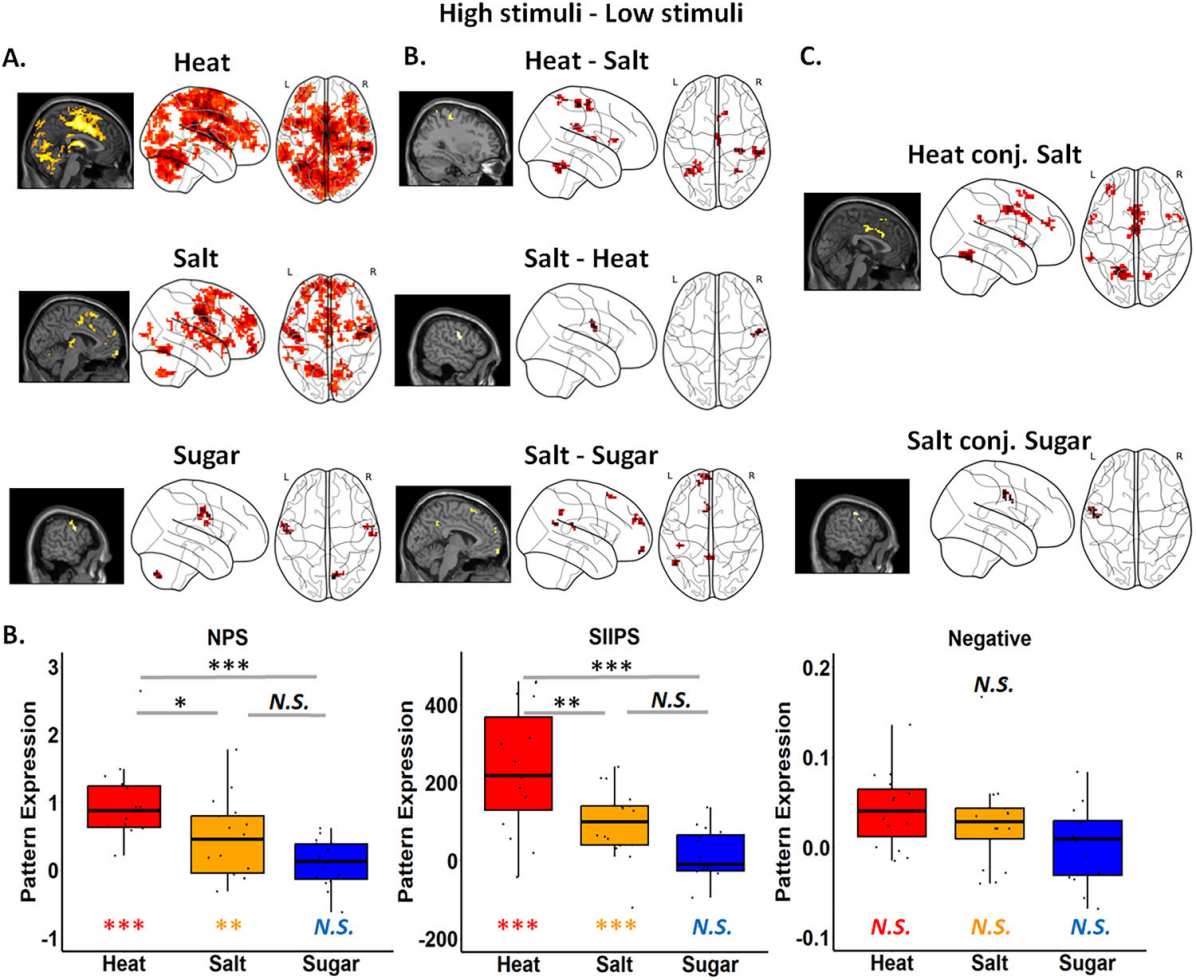
Brain responses to variations in stimulus level. ***A***, Neural activations in response to changes in stimuli (High > Low) for Heat, Salt, and Sugar groups. The Heat group exhibited the strongest and broadest activations, notably in the midcingulate cortex (ACC/MCC), aIns, posterior insula, and secondary somatosensory gyrus (SII). The Salt group showed key brain activations in the frontal gyrus, insula, thalamus, amygdala, and SII. Activations in both groups were largely consistent with previous studies. The Sugar group demonstrated activations in the IFG, motor cortex, temporal cortex, and SII. ***B***, Group comparisons revealed distinct brain activations for Modality and Aversiveness. For Modality: (1) the contrast of Heat versus Salt showed activations in the supramarginal gyrus, SII, precentral gyrus, frontal gyrus, MCC, parietal lobe, temporal lobe, and cerebellum. (2) The contrast of Salt versus Heat showed significant activations in the postcentral gyrus. For Aversiveness: the comparison of Salt versus Sugar revealed activations in the middle and posterior cingulate cortex, superior medial frontal gyrus, middle temporal gyrus, and SII. ***C***, Conjunction analyses showed shared activations for both modality and aversiveness. For modality, the Heat and Salt groups showed overlapping activations in the ACC/MCC, cerebellum, superior and middle frontal gyrus, temporal pole, right insula, and SII. For aversiveness, the Salt and Sugar groups showed common activations in the precentral gyrus, motor cortex, and SII. ***D***, Neural signature pattern analysis examined three brain patterns. For the two pain-related patterns, NPS (nociception-specific pattern) and SIIPS (non-nociception specific but pain-relevant pattern), both Heat and Salt groups showed significant expression above zero, with Heat exhibiting higher expressions compared with Salt and Sugar. For the Negative affect pattern, which is domain-general, both Heat and Salt groups showed significant expressions, but no group differences were observed. These results indicate domain-specific neural activations for Heat that cannot be entirely attributed to salience or aversiveness. Group differences are marked with black asterisks (significant) or “N.S.” (not significant). Within-group differences (one-sample *t* test) are indicated in the color corresponding to the data bars in the plot. ***p < 0.001; **p < 0.01; *p < 0.05.

Since no between-group activations survived FWE correction, we reported all between-group results in Text 2 and Figure S3. However, to provide a comprehensive overview, we still present the main univariate group comparisons and conjunction analyses in Figure 3.

In supplementary materials, we also asked whether findings could be explained by average perceived intensity or valence, regardless of the group; no voxels survived FWE correction (Text S2).

We used one-way ANOVAs to assess group differences in stimulus-level effects on neural expression [High > Low] for each neural signature pattern (Fig. 3*D*). For NPS and SIIPS, we observed a significant Group effect (NPS, *F* = 11.531; *p* < 0.001; SIIPS, *F* = 15.251; *p* < 0.001), with post hoc comparisons revealing a stronger expression for Heat compared with Salt (NPS, *p* = 0.014; SIIPs, *p* = 0.003) and Sugar (NPS, *p* < 0.001; SIIPs, *p* < 0.001). No significant differences were found between Salt and Sugar (*p*'s > 0.09) for either NPS or SIIPS. For the negative affect pattern, no significant group effect emerged (*F* = 2.458; *p* = 0.097).

To further assess whether the absence of a significant group effect truly reflects a null finding, we conducted a Bayesian factor (BF) ANOVA on the negative affect pattern across the three groups. The results of BF (BF₀₁ = 1.15) suggest anecdotal evidence in favor of the null model over the alternative, indicating weak support for a lack of group differences.

### Common effects of learned cues on OFC activation during anticipation across modalities

**Figure 4.**
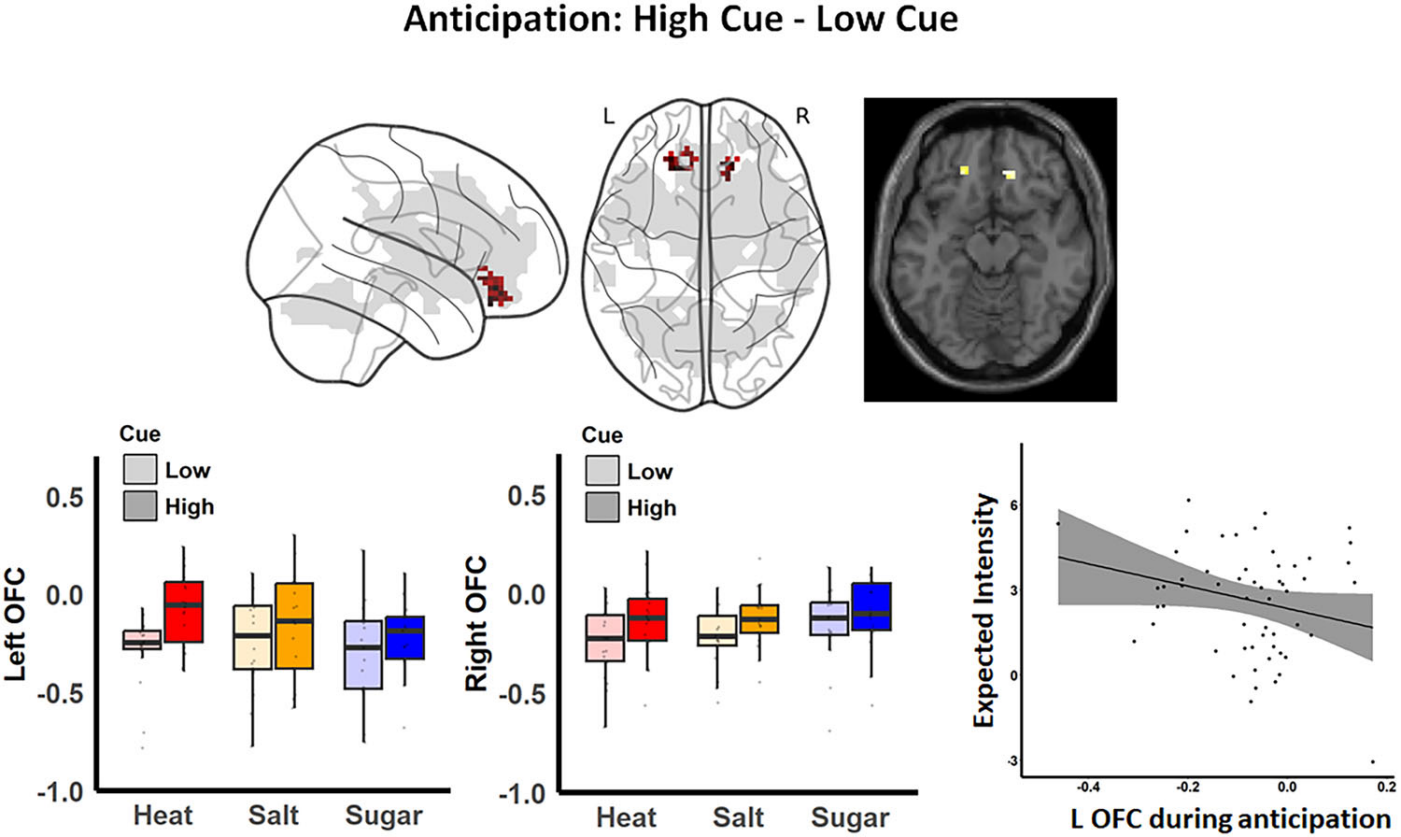
Brain responses to cues during anticipation. Significant activations in the bilateral orbitofrontal cortices (OFC) were observed during anticipation across all groups. These activations (shown in red) overlapped with our previous pain-placebo analgesia mask (shown in gray; Atlas and Wager, 2014), though they did not survive small volume correction. The absence of group differences suggested the domain generality of these activations. Pearson's correlation analysis indicated a trend toward a correlation between cue-related activations in the left OFC and intensity ratings across groups (*r* = −0.25; *p* = 0.063). This evidence further supports the relevance of OFC activation to anticipation, particularly in relation to expected intensity.

Given that changes in the stimulus level strongly shaped perception and brain responses during stimulus outcome and cues paired with these outcomes were associated with differences in expectations, we next tested how cues influenced brain activation prior to outcome delivery, i.e., during anticipation. Whole-brain exploratory analyses revealed activation in the bilateral OFC, with cluster sizes of 25 voxels in the left OFC and 20 voxels in the right OFC (Fig. 4), with no additional activations observed within, across, or between groups. A voxel-level threshold of *p* < 0.001 (uncorrected) was applied, along with a cluster extent threshold of at least 10 contiguous voxels to enhance the robustness of the results. Consistent with our preregistration, we evaluated whether OFC anticipatory activation was correlated with participants’ expected intensity and/or valence. We extracted averaged ROI activations separately for the left and right OFC across all blocks. Spearman correlation analysis revealed a trend toward correlation between cue effects on the left OFC during anticipation and cue effects on expected intensity (*r* = −0.25; *p* = 0.063). No significant correlations were detected for the right OFC or for expected valence ratings [all abs(*r*) < 0.12; *p* > 0.3; Fig. 4]. These findings demonstrate the domain-general engagement of the OFC based on predictive cues during the anticipation of pleasant and unpleasant stimuli, regardless of modality.

### Mediation of common cue effects on subjective intensity and valence

#### Intensity mediation

**Figure 5.**
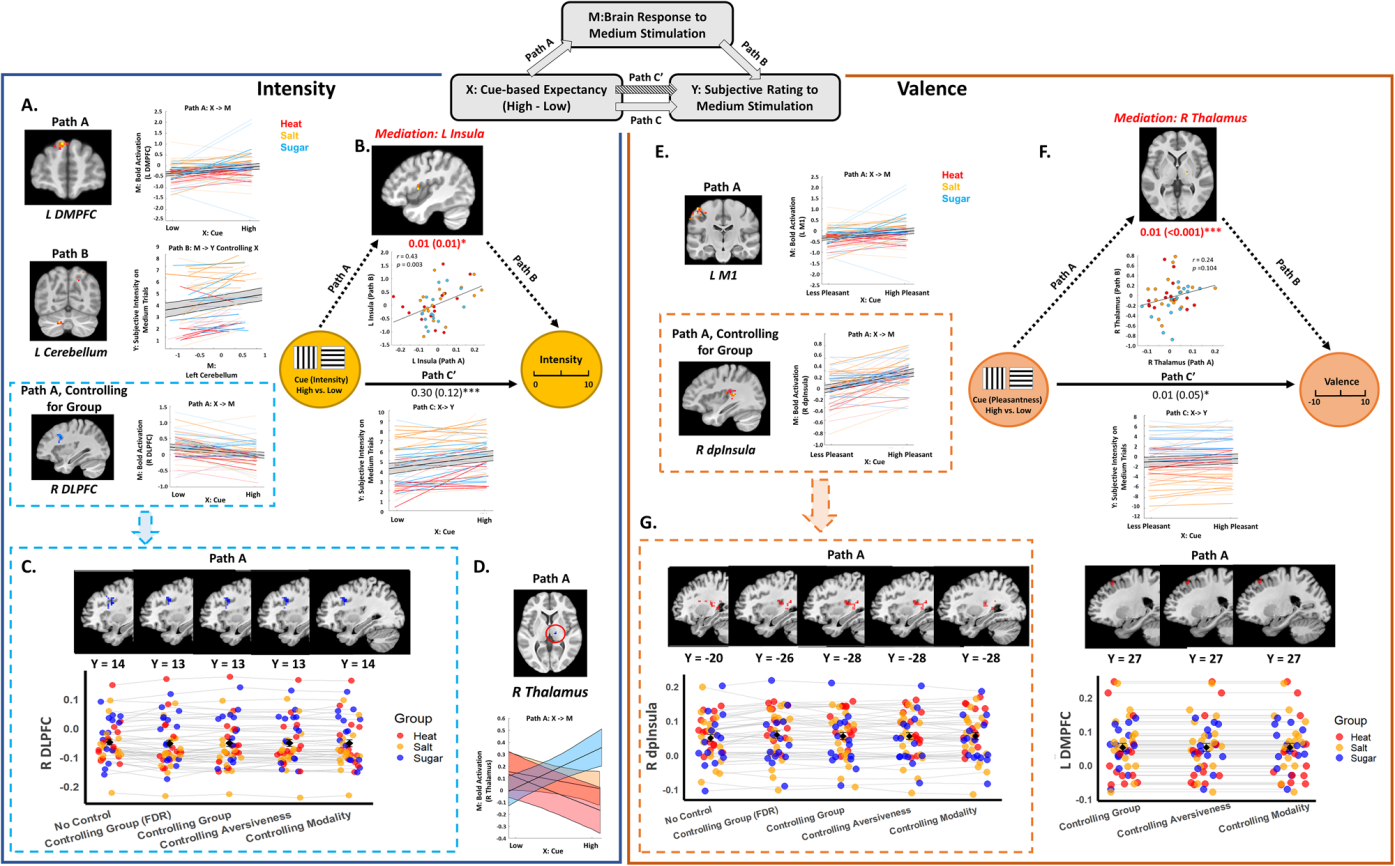
Mediators of cue effects on subjective perception across modalities. This figure presents results of voxelwise multilevel mediation analyses that searched for brain mediators of cue effects on subjective experience (see schematic diagram at the top center). The left panel depicts significant path effects for Intensity as the dependent measure (*Y*), and the right panel illustrates corresponding effects for Valence (Pleasantness). Slope plots below each path diagram illustrate behavioral results (i.e., Path C). ***A***, Path A and Path B effects for Intensity (FDR-corrected). Individual slopes representing each effect are shown on the right, with the black line indicating the average slope across all participants. We identified a positive Path A effect (High Cue > Low Cue) in the left DMPFC, driven by higher DMPFC activation with high cues across all groups and a positive Path B effect in the cerebellum, driven by positive associations between activation and subjective intensity, when controlling for Cue. After controlling for Group, a negative Path A effect emerged in the right DLPFC, driven by higher DLPFC activation on medium trials preceded by low cues relative to high cues. ***B***, Mediators of cue effects on intensity ratings. Whole-brain uncorrected results indicated that the left insula positively mediated cue effects on intensity ratings (Path ab = 0.01; STE = 0.01; *p* < 0.05). Mediation was driven by the covariance between Paths a and Paths b, such that individuals who had stronger cue effects on insula activation on medium trials also had stronger associations between insula and subjective intensity, controlling for cue (see scatterplot). ***C***, Comparison of Intensity path effects with and without moderators. The Path A effect in the right DLPFC remained consistent and robust irrespective of controlling for Group, Aversiveness, and Modality (whole-brain, uncorrected). ***D***, FDR-corrected moderation results. In the right thalamus, the Sugar group exhibited a significantly more positive Path A effect (increased activation on medium trials preceded by High cues) than the Salt and Heat groups, whereas the Heat group demonstrated a significantly more negative effect (larger deactivation on medium trials preceded by High cues) compared with the Salt group. ***E***, Path A and Path B effects for Valence. All results passed FDR correction. A positive Path A effect (cue, high < low for Heat and Salt; high > low for Sugar) was observed in the left premotor cortex (M1), and after controlling for Group, a positive Path A effect was found in the right dorsal posterior insula (dpInsula). Both regions showed higher activation on medium trials preceded by cues that predicted less aversive outcomes. Path B effects did not survive FDR correction; see Table S5 for complete results. ***F***, Mediators of cue effects on subjective valence. FDR-corrected results indicated the right thalamus positively mediated cue effects on pleasantness ratings (Path ab = 0.01; STE < 0.001; *p* < 0.001). Mediation was driven by covariance, such that individuals who had stronger cue effects on thalamus activation on medium trials also had stronger associations between the thalamus and subjective intensity, controlling for cue, although correlations at the cluster level were not significant (see scatterplot). ***G***, Comparison of Valence path effects with and without moderators. The Path A effect in the right dorsal posterior insula and left DLPFC demonstrated similar activations regardless of moderation or control for Group, Aversiveness, and Modality. Positive activations are displayed in warm colors, and negative activations are in cool colors. Dots and lines represent groups: Heat (red), Salt (yellow), and Sugar (blue). Values outside parentheses denote estimated mediation path coefficients, while values inside parentheses indicate standard errors. Significance levels: *p < 0.05; **p < 0.01; ***p < 0.001.

Given that cues affected anticipatory activation and subjective ratings similarly across groups, we next used mediation analysis to isolate brain mediators of dynamic cue effects on subjective perception on medium trials regardless of the group. We first searched for mediators of dynamic cue effects on subjective intensity (Fig. 5*A*; Table 5; Fig. 3*A*). Path *a* in our intensity mediation model isolates brain regions whose response to medium stimuli varies as a function of cue [High vs Low]. Path *a* effects were not associated with significant expression of any signature pattern. Positive Path *a* effects [High > Low] were observed in the left dorsomedial prefrontal cortex (DMPFC) based on whole-brain FDR correction (Fig. 5*A*; Table 5). Whole-brain exploratory analyses revealed positive Path *a* effects in the right medial OFC, left inferior frontal gyrus (IFG), left DMPFC, and MPFC/rostral ACC, and negative path *a* effects [Low > High] in the left superior temporal gyrus, the right IFG, the right angular gyrus, the left postcentral gyrus, the left inferior parietal lobule, and the right DLPFC, among other regions (Table S5; Fig. S3*A*).

**Table 5.** Single-trial mediation results on subjective intensity and valence

		Peak			Voxel size	*Z*	*p*
		*x*	*y*	*z*			
Intensity							
Path *a*	High > low						
	DMPFC (L)	−10	58	38	6	3.55	0.0003
Path *b*	Positive association						
	Temporal pole (L)	−44	−2	−46	1	4.44	<0.0001
	Cerebellum (L)	−16	−68	−34	7	4.41	<0.0001
	Pons	4	−14	−34	1	4.59	<0.0001
	Midbrain	−2	−44	−28	15	4.32	<0.0001
	Superior occipital gyrus (L)	16	−74	52	21	4.79	<0.0001
	Superior occipital gyrus (R)	−10	−74	52	7	6.1	<0.0001
Valence							
Path *a*	High > low						
	M1 (L)	−46	−20	56	46	4.7	<0.0001
	SPL (R)	−10	−46	64	6	5.53	<0.0001
	DMPFC (L)	−14	−2	70	4	4.81	<0.0001
Path *ab*	Positive mediation						
	Thalamus (R)	20	−14	2	2	4.47	<0.0001

Significant activations were presented for each path. All results passed false discovery rate (FDR) corrections. DMPFC, dorsomedial prefrontal cortex; SPL, superior parietal lobule. See uncorrected results in Table S5.

Path *b* evaluates the relationship between brain activation and subjective intensity controlling for cue. We observed significant Path *b* effects on expression of the NPS (*t*_(1,46)_ = 2.15; *p* = 0.037; Mean = 0.19; SD = 0.60) and marginal effects on expression of the SIIPs (*t*_(1,46)_ = 1.96; *p* = 0.056; Mean = 65.7; SD = 230.08) and the negative affect pattern (*t*_(1, 46)_ = 2.01; *p* = 0.05; Mean = 0.02; SD = 0.07). Whole-brain FDR correction revealed positive Path *b* effects in the temporal pole, left cerebellum, midbrain, and bilateral superior occipital cortex (Fig. 5*A*; Table 5). Uncorrected voxelwise results are included in Figure S3*A* and Table S5.

Whole-brain correction did not reveal any significant mediators, nor were mediation effects associated with significant expression of any signature pattern. However, uncorrected results revealed positive mediation by the left anterior/middle insula (Fig. 5*B*; Table S5), in the same region that was previously found to mediate cue effects on subjective pain (Atlas et al., 2010). Extracting from this region revealed that it was driven by covariance between Paths *a* and *b*, such that individuals who showed stronger cue effects on left insula activation (Path *a*) also showed stronger associations between left insula activation and subjective intensity (Path *b*), as visualized in the scatterplot in Figure 5*B*. We also observed negative mediation by the left visual cortex, driven by negative covariance between Paths *a* and *b*, such that individuals who showed stronger cue effects on the visual cortex had more negative associations between activation and subjective intensity, consistent with suppression effects (Fig. S3*A*; Table S5).

#### Valence mediation

Our second mediation model searched for mediators of cue effects on subjective pleasantness while taking group differences in aversiveness into account (see Materials and Methods). Path *a* identified brain regions that showed greater stimulus-evoked activation on medium trials in response to cues that predicted less aversive/more appetitive outcomes, regardless of the group. We observed marginal Path *a* effects on expression of the NPS (*t*_(1, 45)_ = 1.87; *p* = 0.068; Mean = 0.05; SD = 0.19) and the SIIPS (*t*_(1,45)_ = −1.78; *p* = 0.08; Mean = −15.93; SD = 60.56), but no impact on the negative affect pattern (*p* > 0.7). Whole-brain FDR correction revealed positive Path *a* effects in the left motor cortex (Fig. 5*E*), DMPFC, and left superior parietal lobule (Table 5). Exploratory uncorrected analyses additionally revealed positive Path *a* effects in the OFC, amygdala, insula, and DLPFC (Fig. S3*B*; Table S5). No voxels displayed negative Path *a* effects (i.e., greater activation for cues that predicted the more aversive outcome).

Path *b* identifies regions that predict increases in pleasantness on medium trials, while controlling for cue, regardless of the group. Path *b* effects were inversely related to expression of the NPS (*t*_(1, 45)_ = −2.62; *p* = 0.01; Mean = −0.47; SD = 1.22) and marginally related to expression of the negative affect pattern (*t*_(1, 45)_ = −1.88; *p* = 0.066; Mean = −0.03; SD = 0.12). There were no associations with SIIP expression (*p* > 0.5), and no effects survived FDR correction. Uncorrected whole-brain exploratory analyses revealed positive Path *b* effects in the cerebellum, the right hippocampus/amygdala, brainstem, the left Rolandic operculum, the right SMA, and the left visual cortex (Table S5). There were no regions with negative Path *b* effects.

Cue effects on valence were positively mediated by the right thalamus, based on whole-brain FDR correction (Fig. 5*F*; Table 5). Extracting from this region revealed that effects were driven by positive covariance between paths, although correlations were not statistically significant (Fig. 5*F*). Uncorrected analyses additionally revealed mediation by left VLPFC, bilateral DMPFC, right DLPFC, and midbrain in an area near the ventral tegmental area (Fig. S3*B*; Table S5). There were no associations between mediation effects and pattern expression (all *p*'s > 0.2).

In summary, we found significant cue effects and mediation effects for both intensity and valence across groups. These findings suggest domain-general cue effects driven by distinct patterns of brain activation.

### Moderated mediation: evaluating group differences in cue effects on subjective intensity and valence

#### Moderation of intensity effects by group, modality, and aversiveness

We used moderated mediation to determine whether cue effects on medium trials varied across Groups (Pain vs Salt, and Salt vs Sugar), Modality (Heat vs Tastes), or Aversiveness (Aversive vs Appetitive) and to evaluate results when controlling for Group/Modality/Aversiveness. When controlling for Group, we observed negative Path *a* effects in the right DLPFC based on FDR correction (Fig. 5*A*; Table S5); the same region was observed in uncorrected whole-brain exploratory analyses when controlling for Group, Aversiveness, or Modality (Fig. 5*C*; Table 5) or omitting moderators (Fig. 5; Table S5).

We observed moderation of Path *a* effects in the right thalamus by Group (Fig. 5*D*), such that participants in the Sugar group showed greater taste-induced thalamus activation following High cues relative to Low cues, but participants in the Salt and Heat groups showed deactivation with High cues relative to Low cues, and negative Path *a* effects were stronger in the Heat group than the Salt group (Fig. S4*A*). There were no other moderation effects that survived FDR correction. We reported exploratory moderation results of intensity in Text S2.

#### Moderation of group effects for valence

When controlling for Group in our valence mediation model, whole-brain FDR correction revealed significant positive Path *a* effects in the right dorsal posterior insula and left superior parietal lobule (Fig. 5*E*; Table S5). Exploratory uncorrected analyses additionally revealed Path *a* effects in the left DMPFC, right thalamus, and right middle insula (Table S5). Results were similar when we controlled for Aversiveness and Modality (Fig. 5*G*; Table S5).

No other moderation effects survived FDR correction. Exploratory uncorrected moderation results of valence were reported in Text S2.

## Discussion

We tested how learned cues influenced anticipation and perception of pain and hedonic taste at behavioral and neural levels. Cues modulated expectations, subjective intensity, and subjective valence across groups. Cues affected anticipatory activation similarly across domains, with low cues eliciting larger medial OFC deactivation than high cues regardless of modality or aversiveness. Voxelwise multilevel mediation revealed further commonalities: all groups showed right DLPFC deactivation with high cues and stronger right dorsal posterior insula (dpIns) activation with the more pleasant cue. Across groups, dynamic cue effects on intensity were mediated by the left middle insula activity, while the right thalamus mediated cue effects on subjective valence. Moderation analyses revealed limited evidence for domain specificity, with significant group differences only in the posterior right thalamus. Robust domain specificity based on validated neural signature patterns was only evident when examining changes in stimulus intensity rather than cue effects, and we still observed common effects of heat and salt concentration on expression of a negative affect signature (Čeko et al., 2022). As discussed below, these findings indicate that predictive cues modulate perception primarily through domain-general mechanisms.

As stimulation magnitude (temperature or concentration) increased, participants rated higher intensity and unpleasantness (Heat and Salt) or higher pleasantness (Sugar) during both the calibration and fMRI visits. Differences between high- and low-intensity stimuli were accompanied by changes in primary circuits, including pain-related networks for heat and the gustatory cortex for taste that were largely consistent with previous studies (Small et al., 2003; Jensen et al., 2016), supporting the effectiveness of our design. Neural signature patterns revealed both pain-related neural representations and general aversive processing. Relative to salt and sugar, changes in temperature were associated with stronger expression of classifiers for both nociceptive pain (NPS; Wager et al., 2013) and stimulus-independent pain (SIIPS; Woo et al., 2017). Salt concentration also impacted NPS and SIIPS expression, albeit to a lesser extent than temperature. However, changes in sucrose concentration did not affect either pattern. These results indicate that NPS and SIIPS are not merely responsive to nociception or pain but that they respond to stimulus aversiveness more broadly, suggesting they may be sensitive but not specific to nociceptive pain, in contrast to initial findings (Wager et al., 2013; Woo et al., 2017). Importantly, unlike prior work used to validate the NPS and SIIPS (Kross et al., 2011; Atlas et al., 2014; Woo et al., 2017), we compared heat with stimuli that were individually calibrated to be comparable in intensity. This provides a stronger test of pain specificity than when stimuli vary in intensity and salience. Although the stronger expression for heat could reflect higher activation levels, salt was actually perceived as more intense than heat, and the absence of a significant difference between heat and taste in the domain-general negative affect pattern (Čeko et al., 2022) confirms that aversive brain processes were similar across modalities, though the null finding was only weakly supported by evidence from BF analysis. This indicates that heat-related activations reflect nociceptive- and pain-specific processing as well as processes involved in general aversiveness.

Learned cues influenced explicit expectations across all groups. Although salt was expected to be the most intense and sugar most pleasant, cue effects on subjective intensity or valence of moderate stimuli did not differ by group. Anticipatory brain activation also showed no group differences: all groups showed cue effects on anticipation in bilateral medial OFC, consistent with the OFC's role in encoding a general anticipatory value signal (Levy and Glimcher, 2011; Roy et al., 2012), and this activation was marginally associated with individual differences in expected intensity. We note that we measured expectation ratings between blocks rather than every trial to avoid drawing attention to the study's purpose. Thus, we could not measure dynamic associations between expectations and anticipation. Future studies should incorporate trial-level assessments to clarify these relationships.

Our study revealed significant behavioral and neuroimaging evidence for cue effects on perception across sensory domains. Medium stimuli preceded by high cues were perceived as more intense and unpleasant (heat, salt) or pleasant (sugar) than those with low cues. Mediation analyses revealed domain-general cue effects on stimulus-evoked brain activation: regardless of aversiveness, cues modulated prefrontal cortex activation, with high cues increasing left DMPFC and decreasing DLPFC activity relative to low cues. These regions are implicated in cue-based modulation of pain (Atlas and Wager, 2014; Woo et al., 2017; Atlas, 2023) and placebo analgesia, where DLPFC typically increases with expected pain relief (Wager et al., 2004; Atlas and Wager, 2014), consistent with the DLPFC's role in emotion regulation and cognitive control (Kohn et al., 2014; Morawetz et al., 2017). Both regions also contribute to conflict decision-making and value updating (Mitchell et al., 2009), suggesting opposing activation may reflect distinct cognitive processes in updating information.

We accounted for group differences in aversiveness by modeling relative aversiveness/pleasantness. FDR correction showed valence-specific cue effects in right dpIns when accounting for valence and controlling for group, in contrast to our preregistered hypotheses. The dpIns has been considered pain-specific (Segerdahl et al., 2015) and showed pain-specific processing of expectancy (Sharvit et al., 2019). Our findings of similar cue-induced modulation of activation regardless of group suggest that modulation of this region is not specific to pain. All groups showed greater activation with relatively less aversive cue, which is inconsistent with prior work (Atlas et al., 2010) but may be consistent with models implicating dpIns in detecting aversiveness and shifting behavioral strategies (Gehrlach et al., 2019). Accounting for differences in valence also revealed common cue effects in the left DMPFC. Although these regions were sensitive to expected valence, we observed no cue effects on the negative affect signature pattern and found only marginal cue effects on NPS and SIIPS.

Importantly, brain mediators of predictive cue effects on behavioral ratings were largely consistent across domains. Cue effects on intensity were mediated by stimulus-evoked activation in the left anterior/middle insula and left visual cortex, and cue effects on valence were mediated by the right thalamus. All mediators were driven by covariance: stronger cue effects on stimulus-evoked activation corresponded to stronger associations with subjective ratings (positive for the insula and thalamus, negative for the visual cortex). We note that covariance between Paths *a* and *b* was not statistically significant in the thalamus, perhaps due to subtle differences between groups; exploratory analyses indicated significant covariance only in the Sugar group, but we do not make inferences based on the small sample size. The same insula and thalamus regions were previously found to mediate cue effects on pain (Atlas et al., 2010), and the insula region has been shown to be cue-modulated during taste (Nitschke et al., 2006). The portion of the thalamus that we found mediates cue effects on valence contains anatomical projections to the premotor cortex (Behrens et al., 2003; Elsenbruch et al., 2012). The visual cortex has been shown to encode pain-related cue information (Kim et al., 2024) and interact with the attention network potentially engaging gustatory processing (Veldhuizen et al., 2012). These findings suggest that cues engage similar psychological processes to impact behavioral outcomes across different domains, consistent with previous studies showing cross-domain transferability of placebo effects (Zhang et al., 2011; Zhao et al., 2015, 2020).

Our moderation analysis unveiled few domain-specific effects. The only effect surviving correction was a group difference in cue effects on intensity in the right thalamus: high cues increased responses to sucrose and decreased responses to salt and heat, with strongest deactivation observed in heat. This region was near to, but distinct from, the thalamus mediator region referenced above. Signature pattern analysis showed a trend toward moderated mediation on intensity for SIIPS, primarily driven by a more positive mediation effect for Heat than Salt, supporting the specificity of SIIPS in heat (Woo et al., 2017).

This study has several limitations. We were unable to deliver sucrose solutions of comparable high concentration to those used during the calibration visit, which reduced effect sizes and activation in Sugar. Because the highest sugar stimulus was less intense than the highest salt stimulus, direct comparisons between appetitive and aversive stimuli remain inconclusive. Although we found no evidence that group differences in brain activation can be explained by domain-general differences in perceived stimulus intensity or valence, future studies should improve sucrose delivery methods or test alternative rewarding stimuli to ensure matched perception across domains. We used a between-group design to elucidate cue effects across domains while avoiding cross-modal comparisons which could affect decision-making. However, this limited our ability to compare perception and cue effects within individuals and reduced statistical power relative to within-subject designs. Combined with exclusions due to technical issues or movement, this might have impacted the robustness of our inferences, particularly when testing complex interactions or subtle mediation effects. Future studies can use within-subject designs that allow each participant to experience both heat and taste stimuli to enable direct comparisons and increase power, although conclusions may differ if participants engage in relative valuation and find one modality more aversive than another.

Overall, our study demonstrates that expectations derived through uninstructed associative learning can modulate responses across multiple sensory domains during both anticipation and in response to stimuli themselves. Our results suggest that although changes in stimulus intensity affect distinct circuits, learned cues shape anticipation and perception in a largely domain-general way, with the strongest evidence observed for heat and salt. Future studies using improved sugar-delivery methodologies will be important for clarifying whether this effect consistently spans both aversive and appetitive domains, highlighting both shared and unique neural representations of expectation.
